# Preparation and Optimization OF Palm-Based Lipid Nanoparticles Loaded with Griseofulvin

**Published:** 2017

**Authors:** Wen Huei Lim, Yann Jean Tan, Choy Sin Lee, Hui Meng Er, Shew Fung Wong

**Affiliations:** a *Advanced Oleochemical Technology Division (AOTD), Malaysia Palm Oil Board (MPOB), 6, Persiaran Institusi, Bandar Baru Bangi, 43000 Kajang, Selangor, D. E., Malaysia.*; b *School of Postgraduate Studies and Research, International Medical University, No. 126, Jalan Jalil Perkasa 19, Bukit Jalil, 57000 Kuala Lumpur, Malaysia.*; c *Department of Pharmaceutical Chemistry, School of Pharmacy, International Medical University, No. 126, Jalan Perkasa 19, Bukit Jalil, 57000 Kuala Lumpur, Malaysia. *; d *Department of Pathology, School of Medicine, International Medical University, No. 126, Jalan Perkasa 19, Bukit Jalil, 57000 Kuala Lumpur, Malaysia.*

**Keywords:** Lipid nanoparticles, Palm, Griseofulvin, Particle size, Drug loading, Stability, Zeta potential

## Abstract

Palm-based lipid nanoparticle formulation loaded with griseofulvin was prepared by solvent-free hot homogenization method. The griseofulvin loaded lipid nanoparticles were prepared via stages of optimisation, by altering the high pressure homogenisation (HPH) parameters, screening on palm-based lipids and Tween series surfactants and selection of lipid to surfactant ratios. A HPLC method has been validated for the drug loading capacity study. The optimum HPH parameter was determined to be 1500 bar with 5 cycles and among the palm-based lipid materials; Lipid C (triglycerides) was selected for the preparation of lipid nanoparticles. Tween 80 was chosen from the Tween series surfactants for its highest saturated solubility of griseofulvin at 53.1 ± 2.16 µg/mL. The optimum formulation of the griseofulvin loaded lipid nanoparticles demonstrated nano-range of particle size (179.8 nm) with intermediate distribution index (PDI) of 0.306, zeta potential of -27.9 mV and drug loading of 0.77%. The formulation was stable upon storage for 1 month at room temperature (25 ^°^C) and 45 ^°^C with consistent drug loading capacity.

## Introduction

Lipid nanoparticles (LN) was first introduced in 1991 to formulate poorly soluble drugs for better drug protection and a controlled drug release profile (1, 2). The LN was developed from the oil-in-water (o/w) emulsion wherein the oil phase in the o/w emulsion is replaced with solid lipid (3). Investigations have reported the significance of LN for topical use and the results are promising (4, 5). LN forms a coherent film that reduces the transepidermal water loss (TEWL) and thus hydrates the skin to facilitate the penetration of compound from LN (6, 7). It has been reported that the occlusive efficiency of LN is 15-folds higher than that of microparticles (2). 

**Figure 1:**
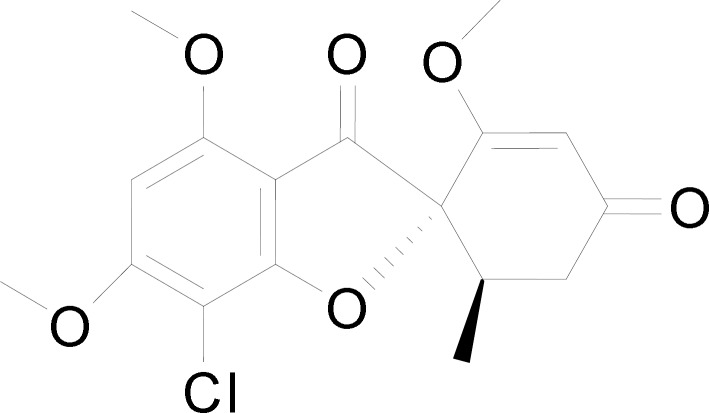
Chemical structure of griseofulvin

Griseofulvin (C_17_H_17_ClO_6_) (Figure 1) was first isolated in 1939 by Oxford from *Penicillium griseofulvum *(8) and it was introduced as an oral antifungal agent (9). It is a fungistatic agent against dermatophytosis (tinea infection) caused by *Epidermophyton*, *Trichophyton* and *Microsporum *by disrupting the mitotic spindle of the dermatophytes and inhibiting its division (10). Till date, griseofulvin is only available in oral preparation form (11, 12) and it is known as a poorly soluble drug with poor absorption via the gastrointestinal tract (13). The absorption can be improved with fatty meal but it requires a long period of oral treatment (10). Griseofulvin has also been reported for its interactions with drugs like coumarin and oral contraceptives (10). In addition, it causes systemic side effects, namely headaches, gastrointestinal disturbance, blood dyscrasis and hepatotoxicity (14). This has led to the interest in developing a topical formulation for griseofulvin. A single topical application of griseofulvin has been reported to have higher skin concentration of griseofulvin (remained detectable from skin after 4 days of application) when compared with a prolonged oral administration of griseofulvin (15). In another study, patients treated with gel form of griseofulvin showed marked clinical and mycological improvement in treating *tinea corporis* (16). Griseofulvin hydrogel added with penetration enhancer such as propylene glycol and N-methyl-2-pyrrolidone also been reported of its anti-fungal activity without causing any skin sensitization (13). Besides, vitamin E-tocopheryl and polyethylene glycol succinate have also been used as penetration enhancer for dermal delivery of griseofulvin (11). 

The *in-vivo* and clinical investigations suggested that it is an effective alternative to oral preparation. Recently, griseofulvin was prepared in glyceryl monostearate LN by microemulsion technique (17) and the formulation demonstrated a prolonged release profile. Besides, griseolfulvin LN was prepared using hydrogenated soybean phosphatidylcholine and dimyristoyl phosphatidylglycerol as lipid core and ethanol as solvent prior to high-pressure homogenizer (18). The freeze-dried griseofulvin LN was reported to have mean particles size of 50 nm. Nevertheless, there is no report on preparation of griseofulvin LN using palm-based lipid materials, though it has been known to be safe on human at high concentrations (19). 

In view of the efficacy of LN in drug delivery for topical formulation and the urge of formulating griseofulvin in topical formulation to overcome the poor drug absorption in gastrointestinal tract and systemic side effects incurred by the oral dosage forms, we aimed to prepare a palm-based LN loaded with griseofulvin using a solvent-free technique. This investigation reports the preparation and optimisation of lipid nanoparticles loaded with griseofulvin by lipid screening, surfactant selections and lipid to surfactant ratios determination. Drug loading capacity study and stability tests of the lipid nanoparticles were conducted for the development of lipid nanoparticles based drug delivery system. 

## Experimental

*Materials and methods*

Palm-based lipids namely Lipid S (palm fatty acid), Lipid G (mixture of palm fatty acid, palm monoglycerides, diglycerides and triglycerides) and Lipid C (palm triglycerides) were provided by Malaysia Palm Oil Board (MPOB). Glycerol, Tween surfactant and methanol (HPLC grade) were obtained from Merck, Germany. Griseofulvin was obtained from Sigma-Aldrich, US. 

*Preparation of Lipid Nanoparticles (LN)*

Lipid phase (blank or with griseofulvin) and aqueous phase: surfactant, glycerol and deionized water were heated to 70^ °^C separately in a water bath. Both phases were then mixed together to form a premix. The premix was subjected to a high-pressure homogenizer (HPH) (Avestin C3, Canada) at different selected cycles and pressures. The mixture was set aside at room temperature for it to recrystallize and form the LN.

*Optimization of High Pressure Homogenisation (HPH) Parameters*

Different types of palm-based lipid such as Lipid S, Lipid G and Lipid C were evaluated for their potential and ease to be prepared into LN. Lipid phase (1% w/w Lipid C, G or S) and aqueous phase: Tween 80 surfactant (1% w/w), glycerol (1% w/w) and deionized water (97% w/w) were used to study the effect of high-pressure homogenizer (HPH) parameters at different cycles and pressures (500-1500 bar). The formulations were subjected to photon correlation spectroscopy (PCS) study. 

*Selection of Lipid and Surfactant*

Formulations were prepared using different lipid (lipid S, lipid G, lipid C) to Tween 80 ratios at the optimum high-pressure homogenization parameter (1500 bar and 5 cycles). The formulations were subjected to photon correlation spectroscopy analysis to select the most compatible lipid to be used in the preparation of LN formulations. After the selection of lipid, the solubilities of griseofulvin in different surfactants (Tween 20, Tween 40, Tween 60, Tween 80) were subsequently determined to select the most suitable surfactant for the preparation of griseofulvin loaded LN. The surfactant [1% (w/w)] was saturated with griseofulvin [0.05%(w/w)] and mixed with deionized water [97.95% (w/w)] and glycerol [1%(w/w)]. All ingredients were heated to 70 ^o^C and mixed together. The premix was subjected to high-pressure homogenization at 1500 bar for 5 cycles. The mixture was then filtered through a 0.22 µM membrane filter to remove the excess griseofulvin. The concentration of griseofulvin in the solution (filtrate) was determined using a high performance liquid chromatography (HPLC) (Agilent 1260, US). 

Subsequently, a series of blank and griseofulvin loaded LN were formulated using the selected lipid and surfactant. All LN was prepared at the optimum HPH parameter (1500 bar, 5 cycles) and at different lipid to surfactant (L:S) ratios, i.e. 1:4, 1:2 1:1, 1:0.5 and 1:0.33. The LN was harvested by ultracentrifugation (Beckman Coulter, US) at 40 K RPM for 3 h. The blank LN and GF-LN were reconstituted in deionized water and subjected to the PCS study. 

*Stability Study*

Griseofulvin loaded LN was analyzed again for the PCS study after one month of storage at room temperature (25 ^°^C) and 45 ^o^C. The harvested GF-LN was also analysed for its drug loading capacity.

*Photon Correlation Spectroscopy (PCS) Study*

Zetasizer Nano ZS (Malvern Instruments, UK) with particle size limitation of 0.3 nm to 10 µM was used in this study. The samples were suitably diluted with deionized water and then subjected to the zetasizer to measure the particle size and polydispersity index. 

*Drug Loading Capacity Study *

Drug loading capacity is the amount of griseofulvin encapsulated by the lipid. Griseofulvin loaded LN was separated from the excess griseofulvin by ultracentrifugation. The formulation was diluted 10 times with deionized water and subjected to ultracentrifugation at 40K RPM for 3 h. After ultracentrifugation, the LN formed the top layer, whereas the free griseofulvin formed a pellet at the bottom of the solution. Meanwhile, the middle portion was a clear solution free of griseofulvin, as confirmed by HPLC analysis. The LN was collected using a micropipette and freeze dried using a freeze dryer (Labconco, US). 

Griseofulvin, if any, present in the lipid nanoparticles was extracted by the following method. The freeze dried LN was weighed and melted at 70 ^°^C until the solid lipid turned into liquid oil. Warm mobile phase (70% methanol) (5 mL) was added to extract the griseofulvin with the aid of sonication (Elmasonic, Germany) for 10 min. In order to separate the lipid from the solvent, the mixture was centrifuged (Eppendorf 5810R, Germany) at 12K RPM at room temperature (25 ^°^C) for 30 min. The supernatant was filtered and the GF content was quantified using HPLC.

The drug loading capacity was calculated using the formula below:

$\mathrm{Drugloading}(\%)=\frac{\mathrm{Amountofextractedgriseofulvin},µg}{\mathrm{Weig}h\mathrm{toflipidnanoparticles},µg}\times 100\%$

The drug loading capacity of the GF-LN formulations was analysed again after one month of storage at room temperature (25 ^°^C) and 45 ^°^C. 

*High Performance Liquid Chromatography (HPLC)*

*Condition.* A modified HPLC method (20, 21) was used for the quantification of griseofulvin. A C18 column was used in the separation. The column was maintained at 25 ^°^C and a mixture of 70:30 methanol and water was used as the mobile phase with a flow rate of 1 mL/min. The mobile phase was filtered with 0.45 µm (nylon) membrane filter and degassed prior to use. Ultra-violet (UV) detector was set at the absorption wavelength of GF at 293 nm. 

*Calibration.* Six known concentrations of griseofulvin were prepared and their HPLC peak areas were determined. A standard stock solution of griseofulvin (100 µgmL^-1^) was prepared by dissolving 5 mg of griseofulvin in 50 mL of mobile phase. Three sets of calibration standards were prepared from the independent stock solutions. The peak areas were plotted against concentrations to obtain the calibration curve. 

*Validation.* For the validation of accuracy and precision of the HPLC method, three quality control (QC) standards at three levels namely low quality control (LQC), middle quality control (MQC), and high quality control (HQC) were prepared. The LQC, MQC and HQC were at 0.9 µgmL^-1^, 12.5 µgmL^-1 ^and 18.8 µgmL^-1 ^respectively. The criterion for accuracy is set to be within the range of 95% to 105%, whereas precision requires a coefficient of variation (CV) within 5% (22). 

*Statistical Analysis*

Statistical analysis was performed using GraphPad Prism version 5.00.

## Results and Discussion

*Optimization of High Pressure Homogenization (HPH) Parameters*

Palm-based lipid was used as lipid core in this study as it also serves as an emollient that soothes and moisturizes the skin especially at fungal infected area, which is commonly unsightly and dries (19). Lipid S, Lipid G and Lipid C were first used to prepare blank LN formulations by varying different cycles and pressures (500-1500 bar) of the high-pressure homogenizer. High-pressure homogenization is a well accepted and widely used method for the preparation of LN (23, 24, 25). The main principles involved are high shear stress and cavitation (26). Lipid S and lipid G formulations were subjected to PCS study. However, the zetasizer was not able to record reading for these formulations as the particles size exceeded the measurement capability of the zeta sizer, stated the presence of big particles. On the other hand, LN formulations prepared from Lipid C were able to be analysed by the zetasizer (Table 1). Statistical analysis using One-way ANOVA followed by Tukey’s post hoc analysis showed a significant reduction (p<0.001) in particle size with increasing pressure and cycle of homogenization. The smallest particle size of 112 ± 5.8 nm was produced at 1500 bar, and 5 cycles of homogenization. It has been reported that high-pressure homogenization setting with 3 to 5 cycles and 500 to 1500 bar are sufficient to produce LN with submicron size (27). The polydispersity index (PDI) of all the lipid C formulations was ranged from 0.263 to 0.443. It has been reported that PDI < 0.05 represents a very narrow distribution (28); PDI < 0.2 represents narrow size distribution (29) and PDI > 0.7 indicates a very broad particle size distribution (28) in lipid nanoparticles. In this study, lipid C produced LN with neither narrow (PDI < 0.05) nor very broad (PDI >0.7) particle size distribution. Cho *et al. *reported on docetaxel-solid LN with neither narrow nor broad particle size distribution (PDI ranging from 0.191 to 0.214) and the LN demonstrated *in-vitro* sustained release profile, enhanced performance in drug absorption and *in-vivo *bioavailability (28). The optimum high-pressure homogenization parameters were therefore standardized at 1500 bar with 5 cycles.

*Macroscopic Evaluation.* Pearling was shown on all lipid S and lipid G formulations prepared with different HPH parameters. When particles tend to arrange in a chain like manner in pearling, their shape transformation is similar to Rayleigh instability (30). The uncontrollable breakage of the pearl structure challenges the stability of the formulation, as reported in a study of an originally oblate lipid vesicle with the insertion of amphiphilic polymer. Pearling has also been reported to be metastable and evolving where they joined to form a larger sphere and intersected to cause destabilization (31). When lipid C was used in formulating lipid nanoparticles, there was no pearling observed in all lipid C formulations.

*Selection of Lipid and Surfactant*

Formulations of lipid G-LN and lipid S-LN again showed pearling at all ratios but not in lipid C-LN formulations (Table 2). The zetasizer also failed to record the particles size of Lipid G-LN and lipid S-LN formulations at all lipid to surfactant ratio stating the presence of big particle. Lipid C formulations at different ratio were recorded in nano-sized with PDI less than 0.5. Therefore, Lipid C was selected for the subsequent studies.

**Figure 2:**
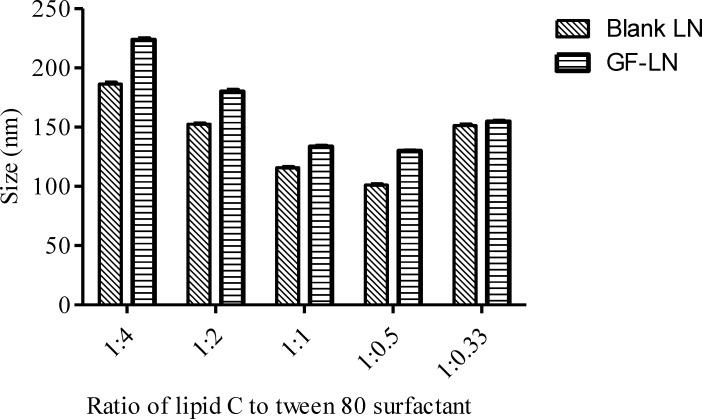
The particle size of the blank and griseofulvin encapsulated lipid nanoparticles prepared by different lipid to surfactant ratio

**Figure 3:**
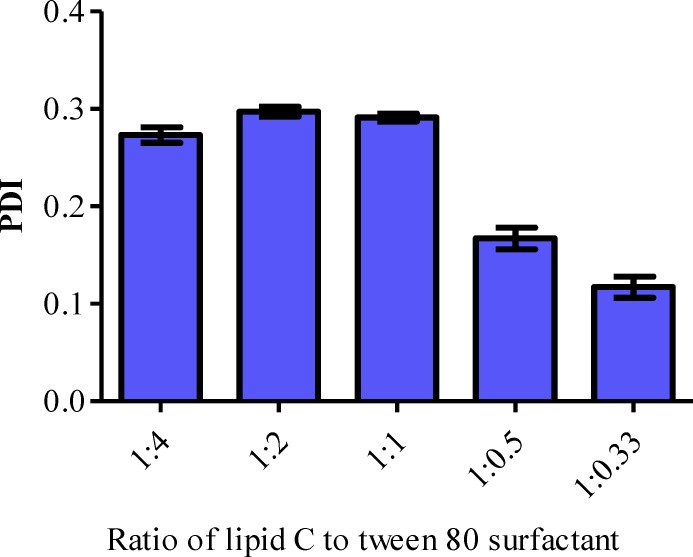
Polydispersity index of blank lipid nanoparticles prepared by different lipid to surfactant ratio. N=3

**Table 1:** The effect of high pressure homogenization on particle size and polydispersity index of lipida nanoparticles. N = 3

**Cycle(s)**	**Pressure (bar)**	**Particle size (nm)** **Mean ± SD**	**Polydispersity index** **Mean ± SD**
3	500	174.9 ± 4.1	0.263 ± 0.006
3	1000	142.7 ± 0.9	0.276 ± 0.009
3	1500	131.4 ± 2.6	0.279 ± 0.007
1	1500	214.5 ± 13.2	0.443 ± 0.041
5	1500	112.0 ± 5.8	0.294 ± 0.006

** Note: lipid** ^a^ **: Lipid C.**

**Table 2:** Macroscopic observation, particle size and polydispersity index of preliminary formulations prepared using Lipid S^a^, Lipid G^b^ and Lipid C^c^.

**Lipid**	**Preliminary** **formulation**	**L:S** **ratio** **d**	**Lipid** **(%)**	**Tween 80 (%)**	**Glycerol** **(%)**	**Deionised** **water** **(%)**	**Macroscopic** **observation**	**Particle** **size (nm) Mean ±** **SD**	**Polydispersity** **index** **Mean ± SD**
**Lipid S**	PS0	1:0	1	-	-	99	Pearling	-	-
	PS1	1:4	0.25	1	1	97.75	Pearling	>10000	>10000
	PS2	1:2	0.5	1	1	97.5	Pearling	>10000	>10000
	PS3	1:1	1	1	1	97	Pearling	>10000	>10000
	PS4	1:0.5	2	1	1	96	Pearling	>10000	>10000
	PS5	1:0.33	3	1	1	95	Pearling	>10000	>10000
**Lipid G**	PG0	1:0	1	-	-	99	Pearling	-	-
	PG1	1:4	0.25	1	1	97.75	Pearling	>10000	>10000
	PG2	1:2	0.5	1	1	97.5	Pearling	>10000	>10000
	PG3	1:1	1	1	1	97	Pearling	>10000	>10000
	PG4	1:0.5	2	1	1	96	Pearling	>10000	>10000
	PG5	1:0.33	3	1	1	95	Pearling	>10000	>10000
**Lipid C**	PC0	1:0	1	-	-	99	No Pearling	-	-
	PC1	1:4	0.25	1	1	97.75	No Pearling	136.6±4.2	0.462±0.045
	PC2	1:2	0.5	1	1	97.5	No Pearling	138.9±2.8	0.325±0.037
	PC3	1:1	1	1	1	97	No Pearling	112.2±5.4	0.278±0.008
	PC4	1:0.5	2	1	1	96	No Pearling	133.2±0.8	0.153±0.017
	PC5	1:0.33	3	1	1	95	No Pearling	128.1±4.9	0.154±0.018

Note: Lipid Sa: Palm fatty acid.

Lipid Gb: Mixture of palm fatty acid, palm monoglycerides, diglycerides and triglycerides. Lipid Cc: Palm triglycerides.

L:S ratiod: Lipid: Tween 80 surfactant.

**Table 3:** The effect of lipid^a^ to surfactant ratio on photon correlation spectroscopy analysis for blank formulation. N = 3.

**Formulation** **(L:S ratio)**^b^		**Percentage, % (w/w)**			Photon Correlation Spectroscopy Mean ± S		
	**Lipid**	**Tween** **80**	**Glycerol**	**Deionised** **water**	**Size (nm)**	**PDI**	**Zeta potential** **(mV)**
A (1:4)	0.25	1	1	97.75	186.3 ± 3.2	0.273 ± 0.008	-28.7 ± 1.9
B (1:2)	0.5	1	1	97.50	152.6 ± 1.3	0.297 ± 0.005	-27.6 ± 1.8
C (1:1)	1	1	1	97.00	115.7 ± 1.2	0.291 ± 0.004	-27.9 ± 2.0
D (1:0.5)	2	1	1	96.00	101.1 ± 1.3	0.167 ± 0.011	-26.7 ± 1.5
E (1:0.33)	3	1	1	95.00	151.3 ± 2.7	0.117 ± 0.011	-25.7 ± 1.2

a Note: lipid: Lipid C.

b (L:S ratio): Lipid C: Tween 80.

**Table 4:** The effect of lipida to surfactant ratio on photon correlation spectroscopy analysis for griseofulvin lipid nanoparticles. N = 3

**Formulation** **(Lipid:**			**Percentage, % (w/w)**			**Photon Correlation Spectroscopy Mean ± SD**		
**Surfactant Ratio) **	**Lipid**	**Tween** **80**	**Glycerol**	**Griseofulvin**	**Deionised** **water**	**Size (nm)**	**PDI**	**Zeta potential** **(mV)**
A (1:4)	0.25	1	1	0.05	97.70	223.7 ± 3.3	0.306 ± 0.015	-29.0 ± 2.2
B (1:2)	0.5	1	1	0.05	97.45	179.8 ± 4.9	0.306 ± 0.011	-27.9 ± 1.6
C (1:1)	1	1	1	0.05	96.95	133.4 ± 1.9	0.302 ± 0.007	-27.4 ± 2.2
D (1:0.5)	2	1	1	0.05	95.95	129.8 ± 1.0	0.214 ± 0.009	-26.2 ± 1.4
E (1:0.33)	3	1	1	0.05	94.95	154.7 ± 1.9	0.152 ± 0.008	-25.9 ± 0.9

Note: lipida : Lipid C.

**Table 5:** Stability studies of the lipida nanoparticles at different lipid to surfactantb ratios. N = 3.

**Formulation** **(Lipid:** **Surfactant Ratio)**	**Drug loading capacity** **(%)** **Mean ± SD**					**Photon Correlation Spectroscopy Mean ± SD**												
	Day 1	After 1 month storage at 25 oC		After 1 month storage at 45 oC		Day 1			After 1 month storage at 25 oC					After 1 month storage at 45 oC				
						Size (nm) Mean ±SD	PDI Mean ±SD	Zeta potential (mV) Mean ±SD	Size (nm) Mean ±SD	PDI Mean ±SD		Zeta potential (mV) Mean ±SD		Size (nm) Mean ±SD	PDI Mean ±SD		Zeta potential (mV) Mean ±SD	
**A (1:4)**	0.672		0.670		0.664	223.7	0.306	-29.0	0.664		0.670		223.7	-29.0		0.670		0.306
	±0.016		±0.008		±0.048	±3.3	±0.015	± 2.2	±0.048		±0.008		±3.3	± 2.2		±0.008		±0.015
**B (1:2)**	0.772		0.770		0.770	179.8	0.306	-27.9	0.770		0.770		179.8	-27.9		0.770		0.306
	±0.028		±0.008		±0.013	±4.9	±0.011	±1.6	±0.013		±0.008		±4.9	±1.6		±0.008		±0.011
**C (1:1)**	0.724		0.716		0.711	133.4	0.302	-27.4	0.711		0.716		133.4	-27.4		0.716		0.302
	±0.029		±0.010		±0.017	±1.9	±0.007	±2.2	±0.017		±0.010		±1.9	±2.2		±0.010		±0.007
**D (1:0.5)**	0.624		0.616		0.586	129.8	0.214	-26.2	0.586		0.616		129.8	-26.2		0.616		0.214
	±0.018		±0.032		±0.010	± 1.0	±0.009	± 1.4	±0.010		±0.032		± 1.0	± 1.4		±0.032		±0.009
**E (1:0.33)**	0.441		0.424		0.386	154.7	0.152	-25.9	0.386		0.424		154.7	-25.9		0.424		0.152
	±0.010		±0.014		±0.030	±1.9	±0.008	± 0.9	±0.030		±0.014		±1.9	± 0.9		±0.014		±0.008

Note: lipida: Lipid C. surfactantb: Tween 80.

Tween (polysorbate), a non-ionic surfactant is known to be a milder irritant than the anionic and cationic surfactants (32). Surfactant or surface tension reducing agent is able to stabilize an emulsion. It can also act as a co-solvent that improves the entrapment of drug in the lipid nanoparticles (33). Tween surfactant is widely recognized as a safe surfactant in the drug delivery formulation (34, 35). Hence, Tween series surfactants (Tween 20, 40, 60 and 80) with increasing alkyl chain length were used in this study. It has been reported that drug solubility improves with increasing alkyl chain length of the Tween surfactant if the solubility takes place in the core of the micelle structure (36). Nevertheless, the solubility of griseofulvin did not demonstrate an obvious trend of increment with the increase of alkyl chain length of the Tween surfactants. The solubility of griseofulvin in Tween 20, 40, 60 and 80 were recorded as 47.5 ± 2.29 µgmL^-1^, 50.1 ± 2.15 µgmL^-1^, 44.2 ± 1.82 µgmL^-1 ^ and 53.1 ± 2.16 µgmL^-1 ^respectively. The solubility of griseofulvin could happen in between the hydrophobic core, the core-surface interface of the micelles and in the palisade layer (36). As reported, non-ionic surfactants such as Tween surfactant can facilitate solubility at the palisade layer. Among all the Tween surfactants, the saturation solubility of griseofulvin in Tween 80 showed the highest concentration of griseofulvin and therefore was selected to be used in the studies. 

*Blank LN.* Blank LN formulations were prepared by different ratios of lipid C and Tween 80 surfactant and subjected to PCS analysis. The data were analysed using One-way ANOVA followed by Tukey’s post hoc analysis (Table 3). In LN formulations, higher concentration of surfactant is associated with smaller particle size (37). This was observed in the preparation of blank LN formulations D (Lipid: Tween 80 surfactant=1:0.5) and E (Lipid: Tween 80 surfactant=1:0.33), whereby the increase in the lipid concentration from 2% to 3% resulted in a significant (p<0.001) increment in the particle size of the lipid nanoparticles (Figure 2). However, beyond the optimum level of the surfactant to emulsify the lipid, there was no further improvement in the particle size (29). The particle size decreased significantly (P < 0.001) from formulation A (Lipid: Tween 80 surfactant = 1:4) to D when the relative amount of surfactant decreased. An increment in the particle size and PDI might be caused by the multilayer on the particle surface formed by the excessive surfactant (38). In addition, the excess surfactants might migrate from the LN interface to form surfactant vesicles, contributing to the increment in particle size (5). This is also in agreement with a report on isotretinoin-loaded LN formulated using stearic acid, glycerylmonostearate, glyceryldistearate, and glyceryldibehnate as lipid phase (39). While maintaining the surfactant (Tween 80) concentration at 5% and increase in the lipid concentration from 7.5% to 10%, the particle size was recorded to be reduced from 359 ± 9 nm to 292 ± 10 nm. However, further increase in the lipid concentration from 5% to 7.5% resulted in an increase of particle size (200 ± 18 nm to 359 ± 9 nm).

The PDI of the formulations A (Lipid: Tween 80 surfactant = 1:4), B (Lipid: Tween 80 surfactant = 1:2) and C (Lipid: Tween 80 surfactant = 1:1) were in the ranged of 0.273, 0.297 and 0.291 respectively. The PDI was significantly decreased when the ratio of lipid and surfactant were further reduced to 1:0.5 (D) and 1:0.33 (E) (Figure 3). According to the Derjaguin-Landau-Verwey-Overbeek (DLVO) theory, the stability of colloidal dispersion is governed by the van der Waals attraction and electrostatic repulsion (40). Hence, the zeta potential of the LN should be as high as possible regardless of its charge, to repel the particles from each other in order to maintain the size and stability. The zeta potential of a stable LN preparation should be at least ± 25 mV (38). It has also been reported that a minimum of ± 30 mV is required for a good physical stability (41). All the blank formulations prepared were categorized as stable since their zeta potential was ranged between -25 mV to around -29 mV (Table 3).

*Griseofulvin loaded LN* ***(***GF-LN)*. *Griseofulvin was incorporated into the LN and it was found that the incorporation of griseofulvin into the LN did not affect the stability of the LN as it did not lead to any significant changes in the zeta potential (Table 4). All of the preparations were in submicron range and their PDI was still maintained below 0.7. Cho *et al.* reported that LN with PDI > 0.7 indicates a broad particle size distribution (28). As expected, the particle size of the lipid nanoparticle increased significantly (p < 0.001) after the incorporation of griseofulvin except for formulation E, in which the particle size remained unchanged at around 150 nm (Table 4 and Figure 2). The PDI of all the griseofulvin loaded formulations were significantly higher than that of the blank formulation but still maintained a PDI of less than 0.7 (p < 0.001), except for formulation B with p < 0.05.

*Stability Study. *The stability of GF-LN upon storage for 1 month at room temperature (25 ^o^C) and 45 ^o^C was monitored (Table 5). There was no significant change in the particle size upon storage except for formulations D and E. There was no significant change in particle size for the latter two formulations after 1-month storage at room temperature (25 ^o^C), but their particle sizes were significantly higher (p < 0.001) after 1-month storage at 45 ^o^C. For PDI, there was no significant difference for formulations A and B after 1-month storage at 25 and 45 °C. However, the PDI increased significantly after storing at 45 ^o^C for formulations C and D (p < 0.001). As for formulation E, the particle size increased significantly upon storage at room temperature (25 ^o^C) (p < 0.05) and 45 ^o^C (p < 0.001). There was no significant difference in the zeta potential of formulations A, B and C upon storage for 1 month at both 25^ o^C and 45 ^o^C. The zeta potential of formulation D was significantly lower (p < 0.001) upon storage at high temperature (45 ^o^C) for 1 month. Meanwhile, the zeta potential of formulation E reduced significantly (p < 0.001) after 1-month storage at both 25 ^o^C and 45 ^o^C. To conclude the stability study of griseofulvin loaded LN, formulations A and B were stable without significant difference in particle size, PDI and zeta potential upon storage for 1 month at 25 ^o^C and 45 ^o^C.

*Drug Loading Capacity Study*

The drug loading capacities of the formulations was determined by the established HPLC method. The results obtained in drug loading study were analyzed with One-way ANOVA followed by Tukey’s post hoc analysis. In this study, excess griseofulvin was added to maximize the incorporation. High-pressure homogenizer was used to reduce the particle size of griseofulvin. Such physical modifications allowed greater surface area to volume ratio thus improved the solubility of griseofulvin (42). Hydrophobicity nature of griseofulvin causes it to favor the lipid phase. Thus, as the lipid concentration increased from 0.25% (Formulation A) to 0.5% (Formulation B), the loading capacity of griseofulvin improved from 0.672 ± 0.016% to 0.772 ± 0.028% (Table 5). Further increase of the lipid content to 1% (Formulation C) led to an insignificant change in the loading capacity of GF (0.724 ± 0.029%). Also, the loading capacity decreased when the lipid concentration increased from 1% (Formulation C) to 3% (Formulation E). This could be due to the increased viscosity that caused a decrease in the efficiency of the homogenizer to further reduce the particle size of griseofulvin to facilitate its solubility and incorporation (43).

Formulation B was chosen as the optimum formulation as its drug loading percentage was significantly the highest (p < 0.01 compared to formulation A; p < 0.001 compared to Formulations D and E). Although its drug loading percentage was insignificantly difference from formulation C, however formulation B consumed a lower percentage of lipid (lesser material). Besides, all of the formulations were stable (no significant change) in terms of drug loading percentage after one-month storage at 25 ^o^C and 45 ^o^C. 

## Conclusion

LN loaded with griseofulvin was successfully prepared from palm-based materials using the simple hot high-pressure homogenization technique. The optimized formulation was obtained from a series of screening and optimisation works including HPH parameters optimization, lipid and surfactant selection and lipid to surfactant ratio optimization in relation to their effects on particle size, formulation stability as well as drug loading capacity. This study suggests that the palm-based triglycerides serve as a potential lipid core of LN for drug delivery. Further studies such as cytotoxicity, antifungal susceptibility tests and skin penetration and retention studies will be reported in another report on the efficacy of the palm-based LN loaded with griseofulvin as topical dosage form.
